# Complete Genome Sequences of Three Fish-Associated *Streptococcus agalactiae* Isolates

**DOI:** 10.1128/genomeA.00025-18

**Published:** 2018-02-08

**Authors:** Anita Jaglarz, Artur Gurgul, William J. Leigh, Janina Z. Costa, Kim D. Thompson

**Affiliations:** a Aquaculture Research Group, Moredun Research Institute, Pentlands Science Park, Penicuik, United Kingdom; b Department of Genomics and Molecular Biology of Animals, The National Research Institute of Animal Production, Balice, Poland

## Abstract

The whole-genome sequences are described here for three group B *Streptococcus* (GBS) (*S. agalactiae*) serotype Ib isolates obtained from tilapia (*Oreochromis niloticus*) farmed at sites in Honduras, Costa Rica, and the United States. The bacteria were isolated from the brains of fish displaying signs of streptococcosis.

## GENOME ANNOUNCEMENT

*Streptococcus agalactiae* is a major cause of streptococcosis in farmed tilapia and has been associated with significant levels of mortality in diseased fish (1, 2). We have selected three group B *Streptococcus* (GBS) (*S. agalactiae*) serotype Ib isolates obtained from the brains of diseased tilapia for complete genome sequencing. The isolates we analyzed belong to clonal complex 552 (CC552) and sequence types 260 (isolates 14-98 and 14-104) and 261 (isolate 14-110) and were typed using molecular serotyping and multilocus sequence typing (MLST), as we previously described for serotype Ia (3). Serotype Ib isolates belonging to CC552 have a significantly reduced genome size compared to that of other *S. agalactiae* genomes (4) and are predominantly associated with fish and other poikilothermic species (5).

Sequencing of the isolates was performed using a MiSeq system (Illumina, San Diego, CA) with 250-bp paired-end reads. Libraries with an average 550-bp insert were prepared using a TruSeq Nano DNA library preparation kit (Illumina). *De novo* assembly of the reads was performed with the SPAdes version 3.5.0 software (6) and the quality of the assembly assessed with the QUAST software (7). The contigs (>200 nucleotides [nt]) were annotated using the NCBI Prokaryotic Genome Annotation Pipeline to predict protein-coding genes and other functional genome units, including structural RNAs, tRNAs, small RNAs, pseudogenes, control regions, direct and inverted repeats, insertion sequences, transposons, and other mobile elements.

The genomes of *S. agalactiae* Ib isolates 14-98, 14-104, and 14-110 were 1,842,528, 1,815,466, and 1,803,751 bp distributed in 99, 51, and 30 contigs and with average *de novo* assembly coverages of 193×, 227×, and 154×, respectively. The G+C genome content for each of the three isolates was 35.3%. The genome of isolate 14-98 was composed of a total of 1,980 genes, including 1,761 coding genes, 56 RNA genes (50 tRNA), and 163 pseudogenes, while the genome of isolate 14-104 contained 1,910 genes, of which 1,703 are coding genes, 48 are RNA genes (42 tRNAs), and 159 are pseudogenes. The genome of strain 14-110 was composed of a total of 1,883 genes, with 1,647 coding genes, 59 RNA genes containing 52 tRNAs, and 177 pseudogenes.

### Accession number(s).

The genome sequences of *S. agalactiae* strains 14-98, 14-104, and 14-110 have been deposited in the GenBank database under the accession numbers NKQD00000000, NKQC00000000, and NKQB00000000, respectively.
